# A high-throughput approach to identify genomic variants of bacterial metabolite producers at the single-cell level

**DOI:** 10.1186/gb-2012-13-5-r40

**Published:** 2012-05-28

**Authors:** Stephan Binder, Georg Schendzielorz, Norma Stäbler, Karin Krumbach, Kristina Hoffmann, Michael Bott, Lothar Eggeling

**Affiliations:** 1 Institut für Bio- und Geowissenschaften, IBG-1: Biotechnologie, Forschungszentrum Jülich GmbH, D-52425 Jülich, Germany

## Abstract

We present a novel method for visualizing intracellular metabolite concentrations within single cells of *Escherichia coli *and *Corynebacterium glutamicum *that expedites the screening process of producers. It is based on transcription factors and we used it to isolate new L-lysine producing mutants of *C. glutamicum *from a large library of mutagenized cells using fluorescence-activated cell sorting (FACS). This high-throughput method fills the gap between existing high-throughput methods for mutant generation and genome analysis. The technology has diverse applications in the analysis of producer populations and screening of mutant libraries that carry mutations in plasmids or genomes.

## Background

Since the first demonstration of microbial product formation more than a century ago [1], vitamins, antibiotics, nucleotides, amino acids and organic acids have been produced in ever increasing quantities. For example, about three million tonnes of sodium glutamate are produced each year as a small microbial molecule. Bacterial synthesis is increasingly also used for the production of small molecules not naturally made by bacteria, such as pharmaceutical intermediates [2,3] or biofuels [4]. The combination of the successful application of microbial synthesis, progress in synthetic biology and changes in the global economy that necessitate intensified use of renewable raw materials indicates that microbial metabolite production will continue to expand.

Microorganisms are not naturally designed for profitable metabolite formation, however, and there is an unrelenting need to optimize strains and pathways. Current strain improvement strategies make use of a variety of methods for engineering and isolating microbial variants with the desired traits. These techniques fall into two major categories: 'rational' methods, which involve the targeted alteration of known genetic information; and 'random' approaches, which are typically based on the creation of mutant libraries containing nondirected changes in genotype with subsequent screening for phenotypes of interest. Both approaches have been successful but the use of mutant libraries has proven to have distinct advantages. The reason is that the exact genomic mutations necessary to adapt the cellular metabolism for increased product synthesis are often difficult to predict, and that 'rational' methods are restricted to known targets. Random approaches with subsequent screening for the phenotype of interest enable us to overcome these difficulties. They have made possible the commercial-scale production of a variety of compounds, such as the unrivaled formation of succinate by *Escherichia coli *[5] or riboflavin by *Bacillus subtilis *[6]. Random and combinatorial approaches were also profitably used for the development of plasmid-encoded targets for the optimization of pathway flux in *E. coli*. This has been demonstrated with amorpha-4,11-diene production [2], which is an artemisinin precursor that is effective for the treatment of malaria, or with taxadiene production [3], an intermediate of the anticancer compound taxol.

However, with few exceptions, the evaluation of methods that utilize random approaches currently requires the cultivation of individual clones to determine production properties. This presents an obstacle. While high-throughput (HT) techniques for introducing genetic diversity and for product analysis or sequencing are well developed [7], comparable strategies for the identification and isolation of high-producer bacterial cells are still lacking. The opportunity to directly monitor product formation within single cells *in vivo *would add a new dimension to the characterization and development of microbial producers.

Here, we present examples of the monitoring of intracellular metabolite concentrations in single bacterial cells and demonstrate in an HT screen the isolation of new bacterial producer cells, as well as the identification of novel mutations based on whole-genome sequencing. The sensors we use are based on transcription factors (TFs) that regulate the transcriptional output of a target promoter in response to a cytosolic metabolite. Whereas the use of TFs to construct whole-culture biosensors for the detection of environmental small-molecule pollutants has long been established [8], this same approach has remained largely untranslated with respect to single-cell analysis and library screening. TFs are naturally targeted to a variety of small ligands, ranging from amino acids to sugars, sugar phosphates, vitamins, antibiotics, oxoacids and lipids [9]. They can also be engineered to obtain altered specificity [10,11], as recently summarized in a comprehensive review [12]. Coupling transcription of the target gene to a reporter protein provides a molecular device for recognition. This has already been successfully applied for screening in plate-based assays using colony color or colony size [10,13], for instance. Here we make full use of intracellular recognition of a specific metabolite in single cells by applying an autofluorescent protein as reporter and also fluorescence-activated cell sorting (FACS). This enables the isolation in HT screens of new bacterial small-molecule producers with random mutations introduced into the genome that enhance production of the molecule of interest, and we present an example of this.

## Results

### Schematic of approach

The workflow for HT selection of genomic variants of metabolite producers consists of the following steps: a) design of a suitable metabolite sensor, b) generation of genetic diversity in genomes of cells carrying the sensor, c) screening of the mutant library and selection of single producer cells via FACS, d) verification and characterization of mutants, and e) sequencing for target identification. We developed sensors for intracellular detection of basic amino acids, as well as of L-serine and O-acetyl-serine, and demonstrated the feasibility of the approach by isolating bacteria producing L-lysine from a library of randomly mutagenized wild-type (WT) cells, culminating in the identification of new useful mutations by whole-genome sequencing.

### Design of L-lysine sensor

To develop a sensor suitable for intracellular L-lysine detection, we focused on the LysR-type transcriptional regulator (LTTR) LysG of *Corynebacterium glutamicum*. This protein senses elevated concentrations of basic amino acids, causing transcription of its target gene *lysE*, which encodes a basic amino acid exporter [14]. To explore the application of this native regulatory device to the conversion of an intracellular metabolite concentration into an optical output, we characterized the interaction of LysG with its target region upstream of *lysE *in a series of electrophoretic mobility shift assays (Figure S1 in Additional file 1). These data were used to construct the metabolite sensor pSenLys shown in Figure S2 in Additional file 1. It contained *lysG*, together with the LysG-binding site in front of the *lysE *promoter driving transcription of *eyfp *coding for enhanced yellow fluorescent protein (EYFP). In addition, P_tac_-driven *crimson *was incorporated as a second fluorescence protein in the vector backbone. All strains and plasmids are listed in Table S1 in Additional file 1.

### Characterization of L-lysine sensor

**Figure 1:**
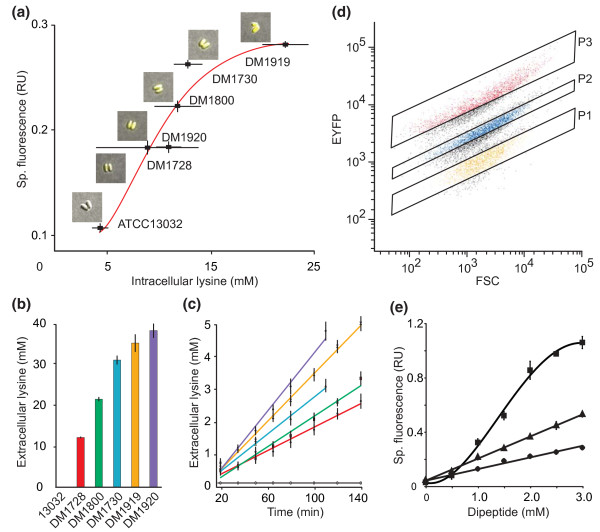
**Characterization of lysine sensor and lysine-producing recombinant cells**. (**a**) Cytosolic L-lysine concentration in *C. glutamicum *WT and five defined lysine producer strains, all carrying pSenLys, and specific fluorescence (Sp. fluorescence) of the cultures. Error bars give the means of three independent cultures for each strain. Fitting the data to the Hill equation describes the signal transfer function by an *n*_app _of 3.19 ± 1.45 and this is shown as the red curve. (**b**) Extracellular accumulation of lysine after 48 h by the strains used in (a). (**c**) Lysine excretion rates of the same strains showing that strains with increased final lysine accumulation have increased excretion rates. Strain DM1920 has two copies of the lysine exporter gene *lysE *present in its chromosome and shows the highest excretion rate, but an intermediate cytosolic lysine concentration (color code as in (b)). (**d**) Differentiation of an equal mixture of cells of ATCC13032, DM1728 and DM1919 each carrying pSenLys by flow cytometry. The success of strain-specific sorting using gates P1 to P3 was over 90%. (**e**) Influence of dipeptide addition on specific fluorescence of *C. glutamicum *WT carrying pSenLys. The peptides Lys-Ala (circle), Arg-Ala (triangle) and His-Ala (square) were added to the cultures at the indicated concentrations. Additionally, Ala-Ala was added to give a total dipeptide concentration of 3 mM. The specific fluorescence was measured 1.5 h after dipeptide addition. FSC, forward scatter; RU, relative units.

The WT of *C. glutamicum *does not excrete L-lysine but there is a genealogy of defined mutants that exhibit increased L-lysine productivity [15]. We determined the cytosolic L-lysine concentration by silicone oil centrifugation and the response of pSenLys in these strains. The WT had a cytosolic L-lysine concentration below 5 mM, while the defined producers had steady-state concentrations ranging from roughly 8 to 25 mM (Figure 1a). A clear increase in specific EYFP fluorescence in cultures is seen with increasing cytosolic L-lysine concentration. As evident from microscopic inspection (insets in Figure 1a), pSenLys is a tool for visualizing cytosolic L-lysine concentrations also within single cells. The range of the cytosolic L-lysine concentration covered translates into a dynamic range of signal output of 8.3-fold.

The fluorescence signal from the pSenLys sensor also correlates with the extracellular L-lysine concentration that accumulates after glucose is consumed (Figure 1b). One instructive exception to this is strain DM1920, which accumulates extracellular L-lysine at concentrations comparable to that of DM1919 despite displaying lower fluorescence due to lower cytosolic concentrations of L-lysine. This is due to altered L-lysine export: Strain DM1919 has one copy of the exporter gene *lysE *and an export rate of 10.1 ± 0.4 nmol minute^-1 ^mg(dry weight) ^-1 ^(Figure 1c), whereas strain DM1920 has two copies of *lysE *(Table S1 in Additional file 1), which results in an increased rate of export of L-lysine of 12.1 ± 0.6 nmol minute^-1 ^mg(dry weight) ^-1^, and thus reduced cytosolic concentration. This observation provides an opportunity of influencing the read-out properties of a sensor that had not previously been taken into consideration. As demonstrated for the production of antibiotics [16], amino acids [17], and biofuels [4], small-molecule production depends on export proteins. The ability to manipulate export activities permits cytosolic concentration of the substrate to be increased or decreased, which may be helpful, for example, when using strains that display high productivity and high cytosolic concentrations of substrate.

To isolate single cell producers by HT flow cytometry, it is essential that the cells can be separated according to their fluorescent properties. To demonstrate that this is the case with pSenLys, sensor-carrying cells of WT, DM1728, and DM1919 grown in glucose minimal medium were mixed in a 1:1:1 ratio to give a total of 4 × 10^7 ^cells ml^-1^. This cell population was analyzed via FACS at a rate of 10^3 ^events per second. Clear differentiation of the population was achieved on the basis of intensity of the EYFP signal (Figure 1d). Three further qualities were assessed (Table S2 in Additional file 1): 1) the sorting specificity achieved using gates P1 to P3 resulted in the selection of ≥ 89% of the L-lysine producer expected within the respective gate; 2) the recovery of viable single cells for each gate was ≥ 84%; 3) when DM1728 was mixed with a 10,000-fold excess of WT cells and then re-isolated, 92% of the prepared cells were DM1728.

LysG recognizes L-arginine and L-histidine in addition to L-lysine [14]. Peptide addition is a proven method to increase the cytosolic pool of a specific amino acid in *C. glutamicum *or *E. coli *[18,19]. The fluorescence response of *C. glutamicum *carrying pSenLys to exogenously applied Lys-Ala is similar to endogenously synthesized L-lysine (Figure 1e). The fluorescence response due to Arg-Ala supply was substantially greater, and that to His-Ala was greater still, which may indicate a higher affinity of these ligands for LysG.

### Further metabolite sensors

**Figure 2:**
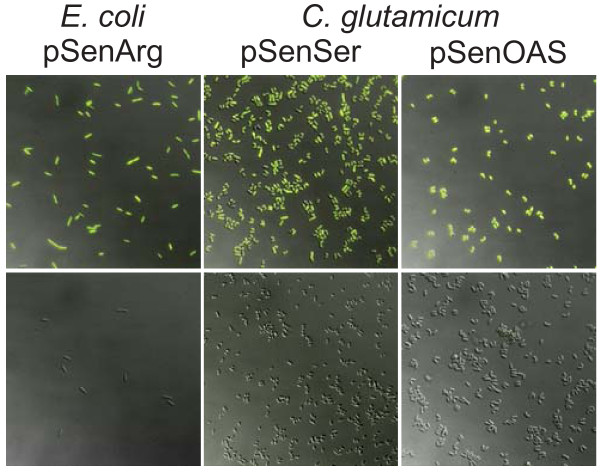
**Metabolite sensors reporting on cytosolic arginine, serine, and *O*-acetyl-serine**. Cells of *E. coli *DH5α (left) carrying pSenArg fluoresce when Arg-Ala was added (top), but not following supplementation with Ala-Ala (bottom). The peptide-dose response curves for Arg-Ala, Lys-Ala and His-Ala are shown in Figure S3 of Additional file 1. The serine-producing strain *C. glutamicum*-Ser4 (middle) [45] with pSenSer is fluorescent (top), but the WT carrying pSenSer is not (bottom). (**c**) Fluorescence is seen with the L-cysteine producer *C. glutamicum*-Cys3 carrying pSenOAS (top right), but not with the control strain (bottom right). Epifluorescence microscopic analysis was done at λ_ex _= 490 to 510 nm and λ_em _= 520 to 550 nm.

To assess the general utility of TFs for reporting on small-molecules at the single-cell level, we constructed pSenArg based on ArgP of *E. coli *controlling *argO *transcription as a function of cytosolic L-arginine [20]. When pSenArg was assayed for dipeptide responsiveness, the addition of Arg-Ala resulted in fluorescent *E. coli *cells (Figure 2, left), with fluorescence increasing in proportion to the dose of dipeptide (Figure S3 in Additional file 1). As additional metabolite sensors, pSenSer and pSenOAS were constructed (Table S1 in Additional file 1), suitable for L-serine (Figure 2, middle), and *O*-acetyl-L-serine detection in *C. glutamicum *(Figure 2, right), respectively.

### Generation of genetic diversity and library screening

To demonstrate the feasibility of metabolite sensors for HT screening of mutant libraries, we introduced chromosomal mutations into the WT of *C. glutamicum *carrying pSenLys by treatment with MNNG (N-methyl-N'-nitro-N-nitrosoguanidine), one of the most effective chemical mutagens for creating genetic diversity [21]. While the separation of mixtures of defined producers could be achieved using FACS (Figure 1d), direct processing of the mutagenized library with FACS was not successful. We used a liquid culture recovery and enrichment step taking into account that metabolically active cells require 2 h for Crimson synthesis (Figure S4 in Additional file 1), and that cells derived from the glycerol stock have to be incubated for 6 h on minimal medium to establish host-specific EYFP fluorescence. The suspension of mutagenized cells from glycerol stock (200 μl) was diluted into minimal medium containing IPTG (isopropyl-β-D-thiogalactopyranoside and, after 2 h, 6.5 × 10^6 ^Crimson-positive cells were sorted in minimal medium. After cultivation for a further 22 h, 350 EYFP positive cells were spotted onto minimal medium plates. Of these, 270 grew into colonies within 48 h.

### Producer verification

**Figure 3:**
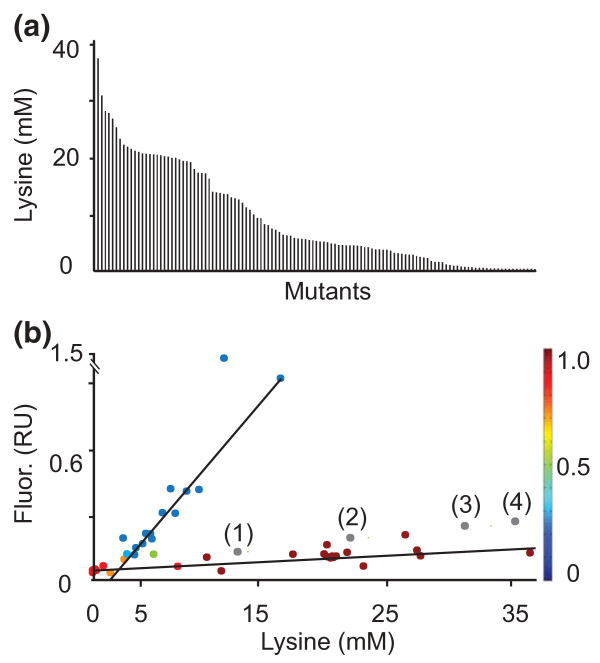
**Characterization of L-lysine-producing mutants isolated by FACS from a library of chemically mutagenized wild type cells**. (**a**) The spectrum of L-lysine accumulation in the culture supernatant of 120 mutants obtained using FACS selection. Mutants were grown in minimal medium with 4% (w/v) glucose and lysine concentrations determined after 48 h. (**b**) Specific culture fluorescence was determined for 40 arbitrarily chosen mutants. Two clusters are apparent after applying an expectation maximization (EM) algorithm to construct a distribution containing maximum likelihood estimates of the parameters in a Gaussian mixture model with two components for data in the 40-by-2 data matrix [22]. The heat bar on the right gives the probability of clones belonging to cluster one, which corresponds to that with the flat curve. The probability of clones belonging to cluster two, corresponding to that with the steep curve, uses the same heat bar with the highest probability in blue and lowest in red. The four gray circles marked (1) to (4) give lysine accumulation and specific fluorescence for the defined recombinants DM1728 (1), DM1800 (2), DM1730 (3), and DM1919 (4) used in Figure 1a. RU, relative units.

The 270 colonies were used to inoculate microtiter plates, to enable HT screening of cultures using 0.75 ml minimal medium. L-lysine was detected in culture supernatants of 185 clones. Re-cultivation of 120 clones (Figure 3a) revealed that the L-lysine concentration ranged from 0.2 to 37 mM. Four clones accumulated 3.6 to 5 mM L-lysine plus 0.6 to 0.8 mM L-arginine and one clone 24.9 mM L-lysine plus 0.6 mM L-arginine. None of the mutants accumulated L-histidine, possibly due to the length of this pathway and its tight regulation.

Of the 120 mutants, 40 were selected randomly, and their culture fluorescence and growth recorded (Figure S5 in Additional file 1). Using an expectation maximization algorithm [22], two clusters relating specific fluorescence to L-lysine accumulation were apparent (Figure 3b). The cluster represented by the lower curve showed characteristics similar to the defined recombinant strains used in Figure 1a, which are included in Figure 3b as gray dots and numbered in parentheses. This cluster includes the mutants of main interest. Mutants in the other cluster show in part extreme fluorescence at a comparatively low extracellular L-lysine accumulation. Since we screened for high fluorescence, it is possible that mutants with reduced L-lysine export activity - and therefore increased intracellular L-lysine concentration - accumulated. This finding warrants further exploration. Cellular export activity is influenced by a number of parameters, including the lipid environment of carriers and the composition of the outer membrane [4,16], which may cause mutants to excrete metabolites at different rates than the WT does.

### Gene analysis in 40 mutants

**Figure 4:**
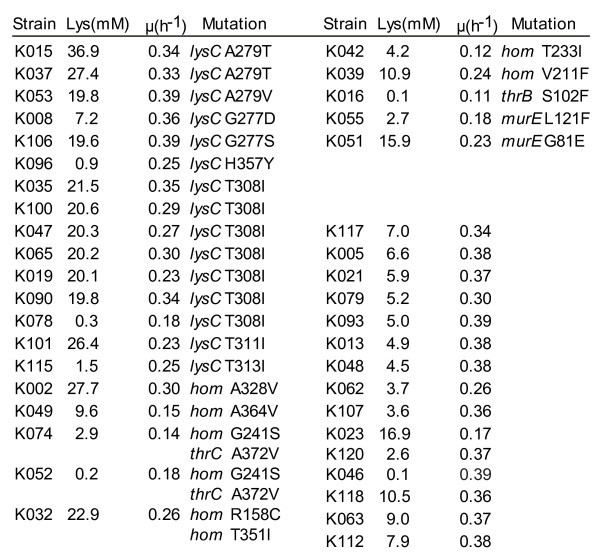
**Genetic characterization of mutants**. In 40 mutants, targeted sequencing revealed mutations in *lysC*, *hom*, *thrC *and *thrB*, with some of the mutations already known from prior work (see text). Mutations that resulted in amino acid exchanges are indicated, along with growth rates and final external lysine titers. The mutation *murE*-G81E in strain K051 was identified by whole-genome sequencing. The second *murE *mutation, *murE*-L121F, was identified by subsequent targeted sequencing. In 15 mutants (plus K051), it was not possible to identify any mutation by site-directed mutagenesis.

**Figure 5:**
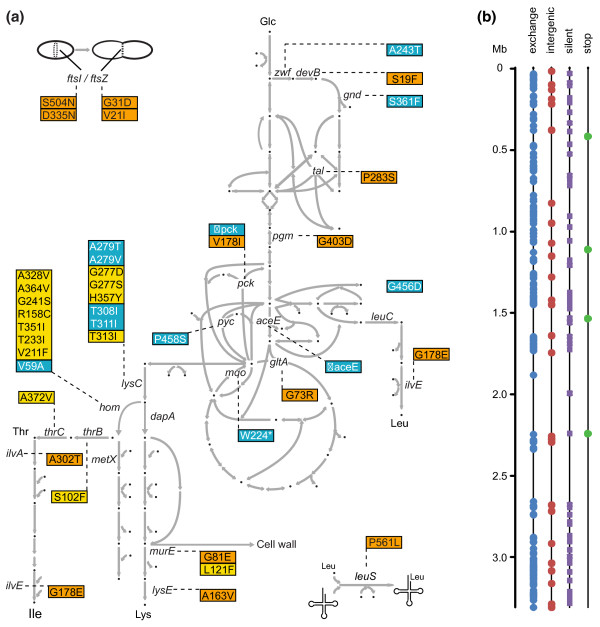
**Sketch of the central metabolism and localization of mutations in *C. glutamicum *together with whole-genome sequencing results of the mutant K051**. (**a**) Pathways and reactions required for and related to lysine synthesis. Genes that carry mutations leading to amino acid exchanges are indicated as follows: blue box, mutations known previously; yellow box, new mutations identified by targeted sequencing in 40 mutants; orange box, new mutations derived from the K051 genome. (**b**) Localization of the 268 SNPs in the genome of strain K051 determined by whole-genome sequencing. The size of the genome is 3.28 Mb, scale on the left. The mutations are classified into those causing an amino acid exchange, those that are silent, those leading to a stop codon, and those located in non-coding regions. The genome sequence of strain K051 is deposited at the European Nucleotide Archive under the accession number HE802067.

We sequenced *lysC*, which encodes aspartate kinase in the 40 mutants described above (Figures 4 and 5a). To date, all L-lysine producers described have a mutation in *lysC*, preventing feedback inhibition of aspartate kinase activity by the concerted action of L-lysine plus L-threonine [23]. In 15 of the mutants that we found, *lysC *was mutated, including seven cases of the known mutation *lysC*-T308I, which is located in the regulatory β-subunit of the aspartate kinase [23]. New mutations - *lysC*-H357Y, *lysC*-T313I, *lysC*-G277D, and *lysC*-G277S - that also affect the regulatory subunit (Figure S6 in additional file 1) were found. We also sequenced *hom *in all 40 mutants, since reduced homoserine dehydrogenase activity reduces L-threonine availability in cells and thus also reduces kinase activity (Figure 5a). Current approaches to engineering L-lysine synthesis rely on just a single *hom *mutation [15]. Seven of the mutants isolated in this study carry novel mutations in *hom *(Figure 4).

In the remaining 18 mutants, neither *lysC *nor *hom *was mutated. We therefore sequenced *thrB *and *thrC *as further genes of L-threonine synthesis (Figure 5a). In strain K016 the mutation *thrB*-S102F was identified and in strain K074 the mutation *thrC*-A342V was found (Figure 4). The introduction of four selected mutations into the WT chromosome resulted in significant L-lysine concentrations (Table S3 in Additional file 1), demonstrating that these new mutations cause increased L-lysine formation.

### Genome sequence of mutant K051

We performed whole-genome sequencing on strain K051, which has no mutation in *lysC*, *hom*, *thrB*, or *thrC *yet accumulates L-lysine up to a concentration of 15.9 mM. Paired-end sequencing on an Illumina HiSeq 2000 provided more than 20 million reads. Trimming and mapping to the WT genome (NC_000913) [15] resulted in a 260-fold coverage (Table S4 in Additional file 1). The genome sequence of strain K051 has been deposited at the European Nucleotide Archive under accession number HE802067. Within K051, 268 SNPs are manifest. They are unevenly distributed in the genome (Figure 5b). The number of SNPs is within the range observed for *E. coli *treated with MNNG [24]. All of the SNPs identified are transitions, as expected with this mutagen, the majority of them resulting in amino acid exchanges (Figure 5b; Table S1 in Additional file 2). In addition, NCgl0863, which carries the amino acid exchange G54D, was partially duplicated, with the variant copy placed 6,108 bp distant from NCgl0863 in an intergenic region.

We searched the mutations in K051 for genes known to increase L-lysine production and to participate in the pathway from glucose uptake up to L-lysine excretion (Figure 5a). Specific mutations in *zwf *and *gnd *in the pentose phosphate pathway are known to increase L-lysine formation due to an increased supply of NADPH [25]; K051 has mutations in *devB *and *tal *that could also be effective. K051 also has mutations in *pck *and *gltA*, genes encoding phosphoenolpyruvate carboxykinase and citrate synthase, where reduced activities are known to increase the supply of pyruvate and oxaloacetate for L-lysine synthesis [26,27]. Also, mutations of branched-chain amino acid metabolism have been demonstrated to increase lysine formation, and K051 carries a mutation in *ilvE*, as well as in the Leu-tRNA synthetase LeuS. Of particular interest was the *murE *mutation (*murE*-G81E) in K051. This gene encodes UDP-N-acetylmuramyl-tripeptide synthetase, an enzyme that utilizes D, L-diaminopimelate as a substrate, as does the D, L-diaminopimelate decarboxylase, in L-lysine synthesis.

### Influence of *murE *mutations on L-lysine synthesis

**Figure 6:**
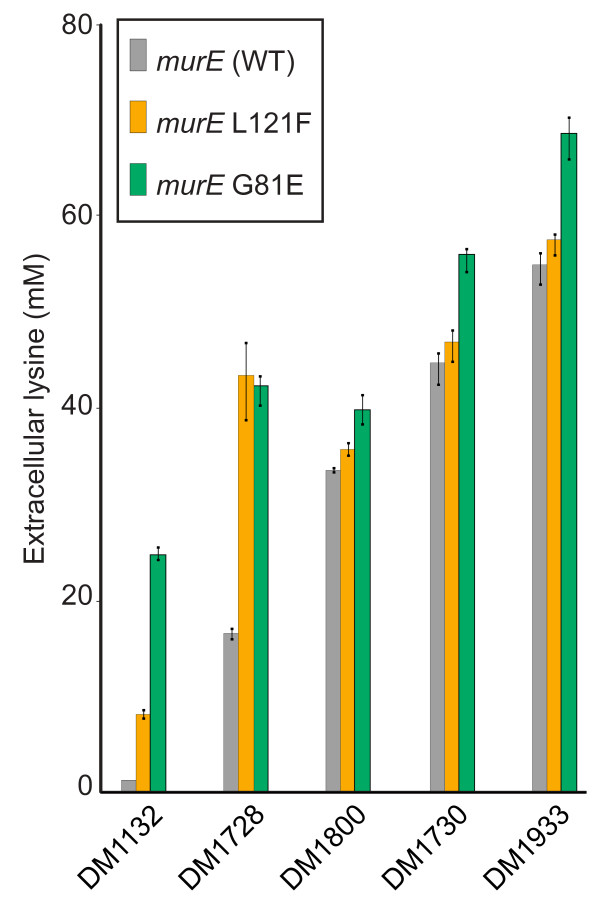
**Effect of *murE *mutations on lysine accumulation**. Lysine production by different strains modified to carry a chromosomal *murE *mutation. Color code: gray, ancestor strains; orange, strains carrying the amino acid exchange L121F in MurE; green, strains carrying the amino acid exchange G81E in MurE.

To determine whether the *murE*-G81E mutation identified could generate increased L-lysine formation, we introduced it by allelic replacement into DM1132, DM1728, DM1730, DM1800, and DM1933. The new strains were cultivated in parallel to their ancestor strains in shake flask cultivations and final L-lysine concentrations were determined after 48 h. As shown in Figure 6, the mutation caused strong L-lysine accumulation when introduced into the WT DM1132 and also DM1728, the strains that have few mutations and which form comparatively little L-lysine. Yet even with the best producer available, strain DM1933, a significant increase in L-lysine accumulation was determined. Given this finding, we sequenced *murE *in the remaining mutants isolated by our HT technology that had no identified mutation (Figure 4), and found *murE*-L121F in strain K055. Introduction of this specific mutation into the five defined L-lysine producers yielded increased L-lysine accumulation, too (Figure 6). Whether the increases with the two *murE *mutations identified were due to increased availability of D, L-diaminopimelate for L-lysine synthesis, or whether a global regulatory effect pushes synthesis of D, L-diaminopimelate remains to be studied.

## Discussion

The key requirement for visualization of single cells with elevated concentrations of a small molecule of interest is the availability of suitable *in vivo *sensor systems with sufficient sensitivity and specificity. There are a large number of options for developing customized reporters sensing intracellular metabolites. They are based on natural molecular recognition, allosteric switching, and gene regulation behavior of proteins and RNA. Every system has its own specific advantages and disadvantages, and the reader is referred to recent reviews on the numerous ideas and ongoing developments in the field [12,28-33]. Whereas protein sensors based on periplasmic binding proteins and Förster resonance energy transfer (FRET) in principle enable concentration determinations in real time, use of TFs relies on expression of the reporter gene. This delay between ligand binding and the corresponding phenotypic change is not a disadvantage in developing or characterizing recombinant cells since stable genetically encoded genotypes are sought. With respect to the use of TFs in metabolite sensing for screening purposes, the present work based on LysR of *C. glutamicum *is the first example where the responsiveness of the optical output to an existing intracellular metabolite concentration is given, and where a TF-based sensor is used in an HT screen applying FACS for the isolation of new bacterial small-molecule producers.

The responsiveness of TFs previously characterized is deduced from the external addition of the effector molecule and whole culture response. Although this may only be of limited significance for screening, it is disadvantageous for precise characterization since various processes such as active uptake, active export, diffusion and degradation of effector might result in a different cytosolic concentration than that present extracellularly. In the case of LysG-based pSenLys, we determined a detection range of 4 to 25 mM intracellular L-lysine. Sensor responsiveness is characterized by an analog-like response that, when fitted to the Hill equation, is described by *n*_app _of 3.19 ± 1.45. It enables the differentiation of WT from medium- and high-level producer cells (Table S2 in Additional file 1). As our intracellular determinations and the comparison of the isogenic strains with one copy and two copies of *lysE *revealed, the effective range of detection may be extended by altering export activity. This could be of relevance for further improvement of good producers. Sensor response and its usefulness will depend on the interplay between the cytosolic concentration of the small-molecule and export activity, as well as on the affinity of the sensor to the effector and target promoter site.

Three of the small-molecule sensors described in the present work are based on a LysR-type TF, and one on a ROK-type TF. Fortunately, the range of small molecules detectable by TFs is large. *E. coli *has more than 230 TFs, with many of them detecting small molecules. In bacteria, TFs have been found to sense sugars, sugar phosphates, vitamins, 2-oxoacids, ions, antibiotics, and acyl-CoA derivatives [9]. Moreover, TFs with new specificities can be generated [11]. An example is AraC, which has been switched from a natural L-arabinose sensor to a sensor detecting D-arabinose [34] or mevalonate [10], and the latter effector specificity has been used in a plate-based assay to screen for improved mevalonate producers. Other sensors that were given new specificities were developed from NahR or XylR for the detection of benzoic acid-related compounds [35], or TetR for structural derivatives of tetracycline [36]. Advances in the design of microbial-based molecular reporters and customizing ligand dependence derived from natural TFs have recently been reviewed [12]. Thus, sensors for a significant number of small molecules of biotechnological or pharmaceutical importance are within reach.

Whereas the WT of *C. glutamicum *does not excrete L-lysine, cytosolic sensing and FACS as an efficient screen enabled the rapid isolation of 185 new mutants accumulating L-lysine in the culture supernatant. The current number of genes where mutations cause increased L-lysine synthesis is about 12 [37,38]. These mutations serve to increase flux through the L-lysine pathway itself, or to increase the pyruvate and oxaloacetate pool, or the NADPH supply. However, there are still unknown mutations to be discovered, since it is known that in an L-lysine-producing mutant developed over decades in classical screenings, many genes of biosynthesis pathways exhibit increased expression [39], and in a similarly derived L-arginine producer, arginine biosynthesis genes are highly expressed in a manner not achievable by plasmid-encoded expression [40]. Our approach provided alleles of known genes, and this is very useful for genomic reconstruction of producers where advantageous mutations are combined, and alleles may result in different productivity [2,41]. The number of 268 SNPs present in K051 is too great to study their individual impact on product formation, but new possibilities might be offered when more genome sequences become available. Striking was the *murE *mutation present in K051. We suggest that the catalytic activity of UDP-N-acetylmuramoyl-L-alanyl-D-glutamate:meso-diaminopimelate ligase in MurE-G81E is reduced, with the consequence that more D, L-diaminopimelate is available for L-lysine synthesis. MurE of *C. glutamicum *is similar to MurE of *Mycobacterium tuberculosis *and *E. coli*, the crystal structures of which are known [42]. From these, it can be deduced that G81E is close to the nucleoside part of UDP-MurNAc-L-Ala-D-Glu, and L121F in the second mutant identified is close to the ATP-binding site. Thus, a reduced activity is meaningful, and in line with the increased L-lysine formation obtained with all strains when the *murE *mutations were introduced in their genomes. It is also in line with the reduced growth rates of these new recombinants (Table S5 in Additional file 1), since less D, L-diaminopimelate is channeled towards cell wall synthesis. An alternative to simple mass balance effects is that a lack of cell wall building blocks initiates a global response that has a positive effect on biosynthesis.

We applied one of our transcriptional sensors for HT screening of a mutant library with chromosomal mutations, but the same principle may also be explored for HT screening of cells carrying plasmid libraries. This is attractive, since many pharmaceuticals currently produced microbially, such as amorpha-4,11-diene, taxadiene and lycopene, use plasmid-encoded biosynthesis pathways, for example, in *E. coli *[2,3,13]. Use of an appropriate sensor combined with FACS-assisted screening may significantly accelerate the development of producers for such small molecules, too. The HT selection routine for mutant isolation closes the gap between HT generation of mutant libraries and HT sequencing technologies, and further applications of sensing small molecules in single cells are in progress, such as the verification of producer population homogeneity and time-lapse microscopy of *C. glutamicum *in microfluidic chips [43].

## Conclusions

This work examines visualization of the intracellular concentration of small molecules at the single cell level by the use of specific TFs. It opens up various possibilities to characterize and analyze single cells in populations with respect to their cytosolic small molecule concentration. We have demonstrated that the visualization of L-lysine combined with HT sorting of genomic mutant libraries via FACS enables the isolation of new mutants. Together with whole-genome sequencing, this therefore establishes rapid access to new mutations to achieve more efficient product formation. In addition to the screening of cells with genomic mutations, the system is also suitable for screening cells with plasmid libraries to identify more efficient product accumulation.

## Materials and methods

### Sensor plasmid construction

The regulatory units were synthesized (LifeTechnologies GmbH, 64293 Darmstadt, Germany) and cloned into pJC1 using the restriction sites *Bam*HI and *Sal*I. An overview of the sensor plasmids is shown in Figure S2 in Additional file 1. The entire plasmid sequences were deposited at EMBL under the accession numbers HE583184 (pSenLys), HE583185 (pSenArg), HE583186 (pSenSer), and HE583187 (pSenOAS1).

### FACS analysis and cell sorting

Cells were diluted to an optical density below 0.1 and immediately analyzed by a FACS ARIA II high-speed cell sorter (BD Biosciences, Franklin Lakes, NJ USA 07417) using excitation lines at 488 and 633 nm and detecting fluorescence at 530 ± 15 nm and 660 ± 10 nm at a sample pressure of 70 psi. Data were analyzed using BD DIVA 6.1.3 software. The sheath fluid was sterile filtered phosphate-buffered saline. Electronic gating was set to exclude non-bacterial particles on the basis of forward versus side scatter area. For sorting of Crimson- or EYFP-positive cells the next level of electronic gating was set to exclude non-fluorescent cells. Background was estimated using non-induced *C. glutamicum *for sorting of Crimson-positive cells. When sorting EYFP-positive cells, non-producing *C. glutamicum *cells were used.

### Mutagenesis and library screening

*C. glutamicum *ATCC13032 carrying pSenLys was grown in 5 ml BHI complex medium (Difco Laboratories Inc., Detroit, MI 48201, USA) containing 25 μg ml^-1 ^kanamycin to an optical density of 5 to ensure exponential growth. Whole-cell mutagenesis was done by the addition of MNNG dissolved in dimethyl sulfoxide (DMSO) to a final concentration of 0.1 mg ml^-1 ^and incubation for 15 mintes at 30°C. The treated cells were washed twice with 45 ml NaCl, 0.9% (w/v), resuspended in 10 ml BHI and regenerated for 3 h at 30°C and 180 rpm. Mutant cells were stored at -30°C as cryostocks in BHI containing 40% glycerol (w/v). Of the initial cells, 46.2% survived the MNNG treatment and among the surviving cells approximately 16% were auxotrophs. For FACS screening, the mutant stock population containing 7.5 × 10^8 ^viable cells per ml was diluted 1:100 in 20 ml minimal medium containing 0.1 mM IPTG to induce expression of the far-red fluorescent protein Crimson, which was taken as an indicator of metabolically active cells. After 2 h of cultivation, 6.5 × 10^6 ^cells were analyzed by FACS and 2 × 10^6 ^Crimson-positive cells collected in fresh 20 ml minimal medium without IPTG. After cultivation for a further 22 h, 1.8 × 10^7 ^cells were screened and 350 EYFP-positive cells spotted on Petri dishes containing minimal medium. Colonies grown after 48 h at 30°C were further analyzed.

### HT cultivation and culture fluorescence analysis

HT cultivation was done in 48-well Flowerplates (FPs; m2p-labs GmbH, 52499 Baesweiler, Germany) at 30°C, 990 rpm and a throw of ø 3 mm. The specific geometry of the FPs ensures high mass transfer performance and can be used together with the microcultivation system BioLector [44], allowing online monitoring of growth and fluorescence. The medium used for FP cultivations was the MOPS-buffered salt medium CGXII [45], with 4% glucose as substrate and 25 μg ml^-1 ^kanamycin to select for maintenance of pSenLys. For offline cultivations, FPs were cultivated on a Microtron high-capacity microplate incubator operating at a shaker speed of 990 rpm, throw ø 3 mm (Infors AG, CH-4103 Bottmingen, Switzerland). Shake flask cultivations were used to compare the consequences of the *murE *mutations for L-lysine accumulation (Figure 4b); these were done in 500 ml baffled Erlenmeyer flasks with 50 ml medium. The medium was the same as used in FP cultivations, except that the phosphate concentration was reduced by half. Cells pregrown in CGXII medium were used as inocula for all cultivations.

### Amino acid quantification

Amino acids were quantified as their *o*-phthaldialdehyde derivatives via high-pressure liquid chromatography using a uHPLC 1290 Infinity system (Agilent, Santa Clara, CA 95051, USA) equipped with a Zorbax Eclipse AAA C18 3.5 micron 4.6 × 75 mm and a fluorescence detector. As eluent, a gradient of 0.01 M Na-borate buffer pH 8.2 with increasing concentrations of methanol was used, and detection of the fluorescent isoindole derivatives was at λ_ex _= 230 nm and λ_em _= 450 nm.

### Determination of cytosolic amino acid concentrations and amino acid export rates

Cells were pregrown as for FP cultivations for 24 h. They were washed once with fresh CGXII medium at room temperature and transferred into new medium in FPs to give an initial optical density of 10, which corresponds to 3.0 mg (dry weight) ml^-1^. Cultures were incubated at 30°C on the Microtron high-capacity microplate incubator as above. Samples were processed at regular intervals to separate extra- and intracellular fluid by silicone oil centrifugation [46]. For the resulting fractions, amino acids were quantified as described above. The intracellular volume used to calculate the internal amino acid concentration was 1.6 μl mg (dry weight)^-1^. When peptides were added (Figure 1e; Figure S3 in Additional file 1) mixtures of di-peptides at a final concentration of 3 mM were used, such as 1 mM Arg-Ala plus 2 mM Ala-Ala, to ensure that a constant supply of Arg-Ala-derived Arg is present over time in the cytosol at the lower Arg-Ala concentrations.

### Epifluorescence microscopic analysis

Fluorescence imaging was performed using a fully motorized inverted microscope (Nikon Eclipse Ti) equipped with a focus assistant (Nikon PFS), Apo TIRF 100× Oil DIC N objective, NIKON DS-Vi1 color camera, ANDOR LUCA R DL604 camera, Xenon fluorescence light source and standard filters for EYFP detection (λ_ex _= 490 to 510 nm; λ_em _= 520 to 550 nm). Differential interference contrast (DIC) microscopy images as well as fluorescence images were captured and analyzed using the Nikon NIS Elements AR software package. Prior to analysis, cells were fixed on soft agarose-covered glass slides.

## Abbreviations

bp: base pair; EYFP: enhanced yellow fluorescent protein; FACS: fluorescence-activated cell sorting; FP: Flowerplate; HT: high throughput; IPTG: isopropyl-β-D-thiogalactopyranoside; MNNG: N-methyl-N'-nitro-N-nitrosoguanidine; SNP: single nucleotide polymorphism; TF: transcription factor; WT: wild type.

## Competing interests

The authors declare that they have no competing interests.

## Authors' contributions

SB performed experimental studies and the FACS analyses. GS constructed sensors and did the graphic work. NS and KH contributed to sensor construction. The determination of pool concentrations and export rates was done by KK, MB contributed to manuscript writing, and LE designed the project and wrote the paper. All authors have read and approved the manuscript for publication.

## Supplementary Material

Additional file 1 **Supplementary Tables S1 to S4 and Figures S1 to S6**. Table S1: strains used. Table S2: quality assessment of sorting cells carrying pSenLys. Table S3: L-lysine formation with mutations introduced by reverse engineering. Table S4: statistics on whole-genome sequencing of strain K051. Table S5: growth rates of *murE *mutants. Figure S1: isolation of LysG and characterization of the LysG binding site. Figure S2: the vector pSenLys and general configuration of sensor plasmids. Figure S3: peptide-dose response curves with sensor-carrying *E. coli *and *C. glutamicum*. Figure S4: development of Crimson and EYFP signals in mixtures of ATCC13032 with DM1728 over time. Figure S5: growth curves and fluorescence of 40 mutant cultures. Figure S6: structural presentation of LysC and localization of mutations identified. Click here for file

Additional file 2 **All mutations of *C. glutamicum *strain K051**. Click here for file
