# Supplementary material for: A high-throughput approach to identify genomic variants of bacterial metabolite producers at the single-cell level
Source: Genome Biol. 2012 May 28;13(5):R40. doi: 10.1186/gb-2012-13-5-r40 (PMC3446293; doi:10.1186/gb-2012-13-5-r40)
Supplement: Additional file 1 — Supplementary Tables S1 to S4 and Figures S1 to S6. Table S1: strains used. Table S2: quality assessment of sorting cells carrying pSenLys. Table S3: L-lysine formation with mutations introduced by reverse engineering. Table S4: statistics on whole-genome sequencing of strain K051. Table S5: growth rates of murE mutants. Figure S1: isolation of LysG and characterization of the LysG binding site. Figure S2: the vector pSenLys and general configuration of sensor plasmids. Figure S3: peptide-dose response curves with sensor-carrying E. coli and C. glutamicum. Figure S4: development of Crimson and EYFP signals in mixtures of ATCC13032 with DM1728 over time. Figure S5: growth curves and fluorescence of 40 mutant cultures. Figure S6: structural presentation of LysC and localization of mutations identified. [file gb-2012-13-5-r40-S1.DOCX]

**Additional file 1**

**Tables and Figures**

**The file contains detailed data on strains and sorting performance as follows:**

| **Name** | **Title** | **Page** |
| --- | --- | --- |
| Table S1 | Strains and plasmids | 2 |
| Table S2 | Quality assessment of sorting cells carrying pSenLys | 3 |
| Table S3 | L-lysine formation with mutations introduced by reverse engineering | 3 |
| Table S4 | Statistics on whole-genome sequencing of K051 | 4 |
| Table S5 | Growth rates of mutants | 5 |
|  |  |  |
| Figure S1 | Isolation of LysG and characterization of LysG binding site | 6 |
| Figure S2 | Vector pSenLys and general configuration of sensor plasmid | 7 |
| Figure S3 | Peptide-dose response with sensor-carrying *E. coli* and *C. glutamicum* | 8 |
| Figure S4 | Development of Crimson and EYFP signals in mixtures of ATCC13032 with DM1728 | 9 |
| Figure S5 | Growth curves and fluorescence of 40 mutant cultures | 10 |
| Figure S6 | Structural presentation of LysC and localization of mutations identified | 11 |
|  | References | 12 |

**Table S1. Strains and plasmids**

| **Strain or plasmid** | **Description** | **Reference** |
| --- | --- | --- |
| WT *C. glutamicum* | WT strain ATCC 13032, biotin-auxotroph | This laboratory |
| *C. glutamicum* DM1132 | WT strain ATCC 13032, biotin-auxotroph | Evonik laboratory |
| *C. glutamicum* DM1728 | *pyc*(P458S), *hom*(V59A) | [[1](#_ENREF_1)] |
| *C. glutamicum* DM1730 | *pyc*(P458S), *hom*(V59A)*, lysC*(T311I), Δ*pck* | Evonik laboratory |
| *C. glutamicum* DM1800 | *pyc*(P458S), *lysC*(T311I) | [[1](#_ENREF_1)] |
| *C. glutamicum* DM1919 | *pyc*(P458S)*, hom*(V59A)*,* 2 copies of *lysC*(T311I), Δ*pck* | Evonik laboratory |
| *C. glutamicum* DM1920 | *pyc*(P458S)*, hom*(V59A)*,* 2 copies of *lysC*(T311I), Δ*pck*, 2 copies of *lysE* derived from WT *C. glutamicum* | Evonik laboratory |
| *C. glutamicum* DM1933 | Δ*pck, pyc*(P458S), *hom*(V59A), 2 copies of *lysC*(T311I), 2 copies of *asd*, 2 copies of *dapA*, 2 copies of *dapB*, 2 copies of *ddh*, 2 copies of *lysA*, 2 copies of *lysE* derived from WT *C. glutamicum* | [[2](#_ENREF_2)] |
| *C. glutamicum*-Ser4 | ATCC13032 Δ*sda*AΔ*pab*ABC pserAfbrCB | [[2](#_ENREF_2)] |
| *C. glutamicum*-Cys3 | ATCC13032 Δ*sda*AΔ*pab*ABCΔ*aec*D pserAfbrCB | This laboratory |
| *E. coli* DH5α | F- endA1 glnV44 thi-1 recA1 relA1 gyrA96 deoR nupG Φ80dlacZΔM15 Δ(lacZYA-argF)U169, hsdR17(rK- mK+), λ– | [[3](#_ENREF_3)] |
| pK19mobsacB | Km^r^, Suc^r^, mobilizable (*oriT*), *oriV* | [[4](#_ENREF_4)] |
| pSenLys | Encodes *C. glutamicum* LysG, and its target promoter of *lysE* with a transcriptional fusion to *eyfp* | HE583184 |
| pSenArg | Encodes *E. coli* ArgP, and its target promoter of *argO* with a transcriptional fusion to *eyfp* | HE583185 |
| pSenSer | Encodes *C. glutamicum* NCgl0581, and its target promoter of NCgl0580 with a transcriptional fusion to *eyfp* | HE583186 |
| pSenOAS | Encodes *C. glutamicum* CysR, and its target promoter of *cysI* with a transcriptional fusion to *eyfp* | HE583187 |

The regulatory units of the pSen series of vectors were synthesized (LifeTechnologies GmbH, Frankfurter-Str.129b, 64293 Darmstadt, Germany) and cloned into pJC1. Full sequences have been deposited at EMBL.

**Table S2. Quality assessment of sorting cells carrying pSenLys**

| **Sorting criteria** | **Viability** | | | **Verified strain** | | | **Sorting specificity** |
| --- | --- | --- | --- | --- | --- | --- | --- |
|  | **Sorted in total** | **Grown** | | **WT** | **DM1728** | **DM1919** |  |
| P1 (ATCC13032) | 432 | 386 | 89.4 (%) | 99.4 | 0.6 | 0 | 99.4 (%) |
| P2 (DM1728) | 288 | 257 | 89.2 (%) | 3.1 | 94.8 | 2.1 | 94.8 (%) |
| P3 (DM1919) | 288 | 244 | 84.7 (%) | 4.2 | 6.2 | 89.6 | 89.6 (%) |
| Average |  |  | 87.8 (%) |  |  |  | 94.6 (%) |

| **Sorting criteria** | **Viability** | | | **Verified strain** | | **Sorting specificity** |
| --- | --- | --- | --- | --- | --- | --- |
|  | **Sorted in total** | **Grown (%)** | | **WT** | **DM1728** |  |
| P2 (DM1728) | 200 | 184 | 92.0 (%) | 7.3 | 92.7 | 92.7 (%) |

The upper part of the Table shows the result of sorting the mixture of three strains using gates P1-P3. Viability of sorted cells was determined by counting cfus grown up after spotting single cells on BHI petri dishes and incubating for 48 hrs at 30°C. Strains were verified by cultivating 96 clones in a microtiter plate containing minimal medium CGXII with 4% (w/v) glucose and quantification of their L-lysine forming capability after 48 hrs.

The lower part of the Table shows the result of sorting DM1728 out of 10,000 wild type cells, both carrying pSenLys. Determination of viability and L-lysine formation was as above.

**Table S3. L-lysine formation with mutations introduced by reverse engineering**

| **Mutation** | **Recombinant strain** | **Lysine (mM)** | **Isolated mutant** | **Lysine (mM)** |
| --- | --- | --- | --- | --- |
| *thrB* S102F | Lys016 | 0.4 | K016 | 2.1 |
| *hom* V211F | Lys039 | 6.3 | K039 | 10.9 |
| *hom* A364V | Lys049 | 1.1 | K049 | 9.6 |
| *lysC* H357Y | Lys096 | 0.9 | K096 | 2.3 |

In the recombinant strains the single mutation given in the left column was introduced in the wild type genome ATCC13032 by allelic exchange as described [[4](#_ENREF_4)]. L-lysine accumulations were determined in SK cultivations and the L-lysine formed determined after 48 hrs. For comparison L-lysine accumulations are shown for the original clones from which the specific mutations were derived by targeted sequencing.

**Table S4. Statistical analysis of whole-genome sequencing**

|  | **Strain K051** | |
| --- | --- | --- |
| **Description** | **value** | |
| Number of sequenced reads | 20,156,524 | |
| Avg. length of sequenced read [bp] | 51.0 | |
| Number of reads after trimming | 19,908,254 | |
| Avg. length of reads after trimming [bp] | 48.1 | |
| Number of paired reads after trimming used for mapping | 19,664,448 | |
| Number of reads mapped to reference | 17,877,215 | |
| Coverage (# mapped reads * avg. length/3301500) | 260.5 | |
| SNPs in total | 268 | 100 % |
| Transitions | 268 | 100 % |
| SNPs leading to amino acid exchange | 171 | 63.8 % |
| Silent mutations | 65 | 24.3 % |
| Intergenic SNPs | 28 | 10.4 % |
| Introduced stop-codons | 4 | 1.5 % |

Sequence reads were generated on an Illumina HiSeq 2000 and performed at GATC (GATC Biotech AG, Jakob-Stadler-Platz 7, 78467 Konstanz, Germany). Trimming and mapping was done using the CLC Genomics Workbench Version 4.7.2 software of CLC bio (Finlandsgade 10-12, Katrinebjerg , 8200 Aarhus N, Denmark).

**Table S5. Growth rates of *murE* mutants**

| **Strain** | **µ h^-1^** |
| --- | --- |
| DM1132 (WT) | 0.49 ± 0.11 |
| DM1728 | 0.46 ± 0.16 |
| DM1730 | 0.43 ± 0.11 |
| DM1800 | 0.43 ± 0.16 |
| DM1933 | 0.37 ± 0.13 |
| DM1132 (L121F) | 0.45 ± 0.09 |
| DM1728 (L121F) | 0.43 ± 0.20 |
| DM1730 (L121F) | 0.40 ± 0.11 |
| DM1800 (L121F) | 0.43 ± 0.19 |
| DM1933 (L121F) | 0.35 ± 0.14 |
| DM1132 (G81E) | 0.39 ± 0.12 |
| DM1728 (G81E) | 0.36 ± 0.16 |
| DM1730 (G81E) | 0.41 ± 0.10 |
| DM1800 (G81E) | 0.45 ± 0.18 |
| DM1933 (G81E) | 0.31 ± 0.21 |

Strains were pregrown for 8 hrs on complex medium BHI, followed by growth overnight in minimal medium CGXII-glucose, and this used to inoculate cultures in a new CGXII-glucose to determine growth rates. Cultivations were done in microtiter plates and growth was recorded from three parallel cultures.


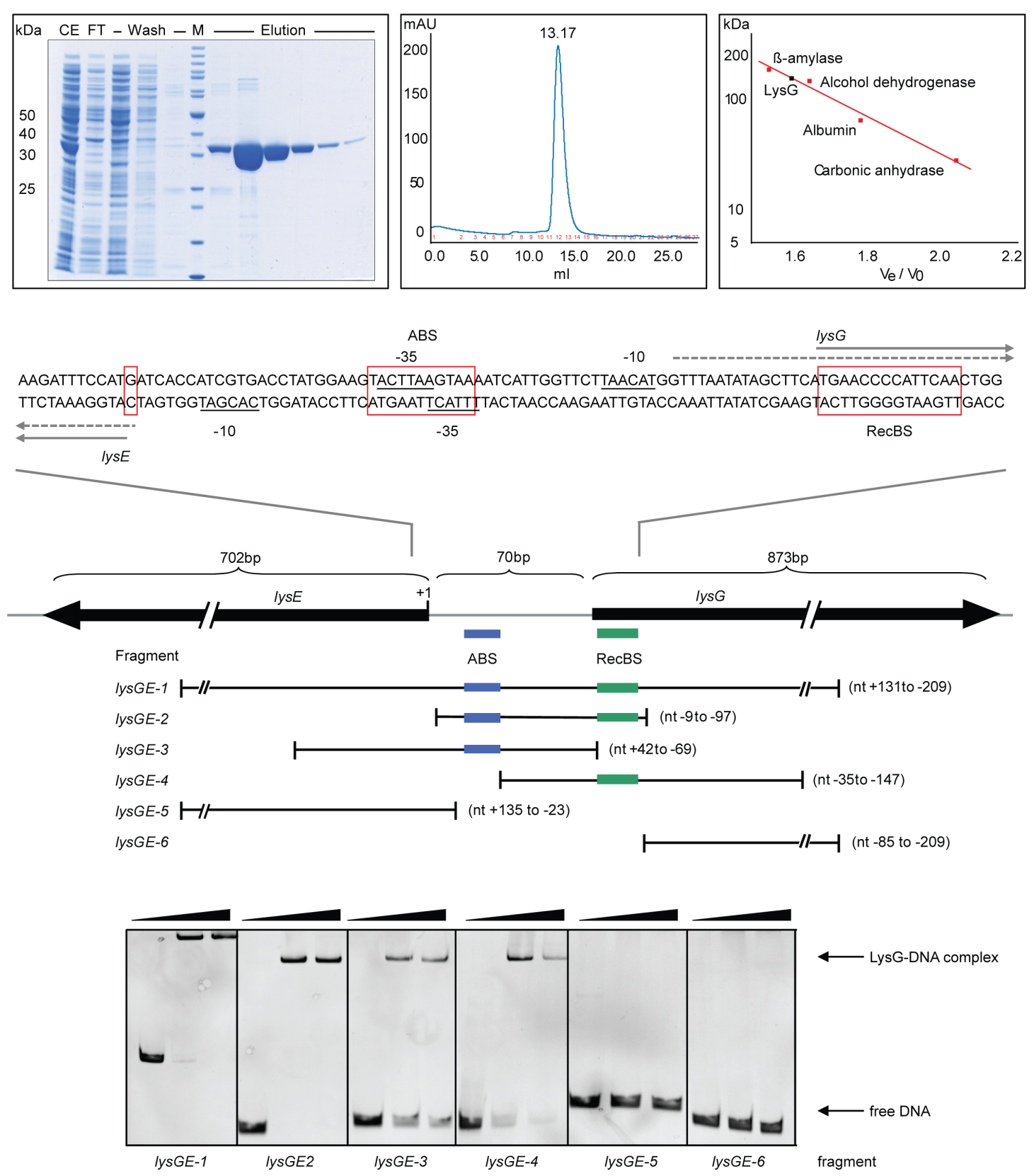


**c**

**b**

**a**

**Figure S1.** Isolation of LysG and characterization of LysG binding site. (**a**) Isolation of His-LysG and gel filtration analysis of LysG devoid of tag together with the calibration curve used for molecular mass determination. LysG eluted with an apparent molecular mass of ~140 kDa. Since the LysG monomer has a molecular mass of 32 kDa, LysG in solution forms a homotetramer similar to other LTTR-type regulators such as CbnR of *Ralstonia eutropha* [[5](#_ENREF_5)] or CysB of *Escherichia coli* [[6](#_ENREF_6)]. (**b**) Overview on *lysGE* organization with the intergenic region on top. The relative position of DNA fragments *lysGE*-1 to *lysGE*-6 are given and the location of the activation binding site (ABS) in blue and the recognition binding site (RecBS) in green. (**c**) Electrophoretic mobility shift assays showing binding of fragments 1-4 to LysG. In each panel (from left to right) no LysG was added, added in a 20-fold excess, and 50-fold excess.

**c**

**b**

a


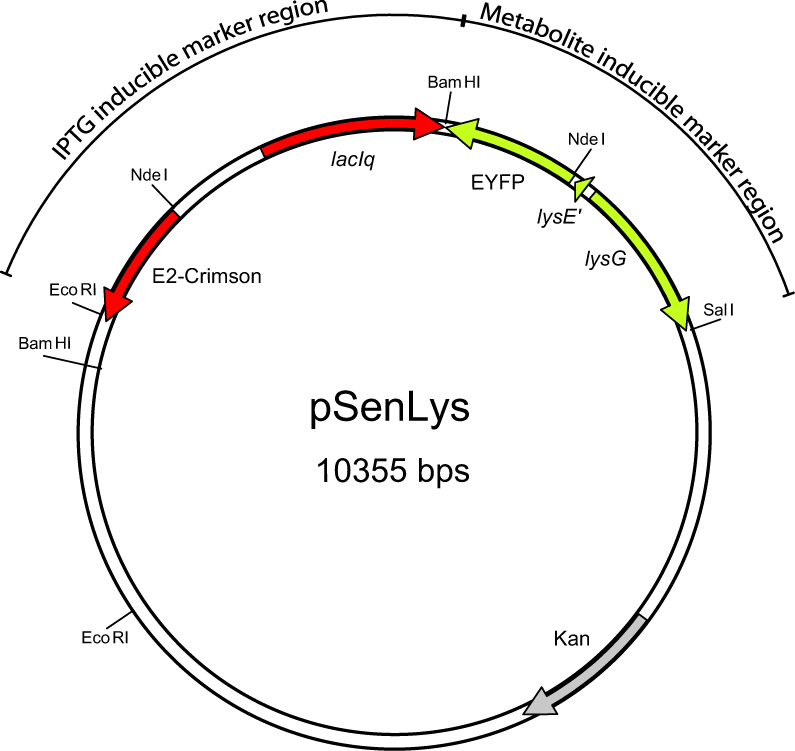


**Figure S2.** Vector pSenLys and general configuration of sensor plasmid. The vector pSenLys is a shuttle vector replicating in both *C. glutamicum* and *E. coli*. It carries the metabolite inducible marker region encoding the L-lysine sensing transcriptional regulator LysG. In presence of L-lysine LysG drives transcription of *lysE*, which is fused with *eyfp,* resulting in green fluorescence. pSenLys also carries the IPTG inducible marker region encoding the LacIq repressor which, in the presence of IPTG, diffuses from the tac promoter region in front of E2-Crimson, resulting in red fluorescence. The system permits the selection of viable cells that are capable of protein synthesis.

**
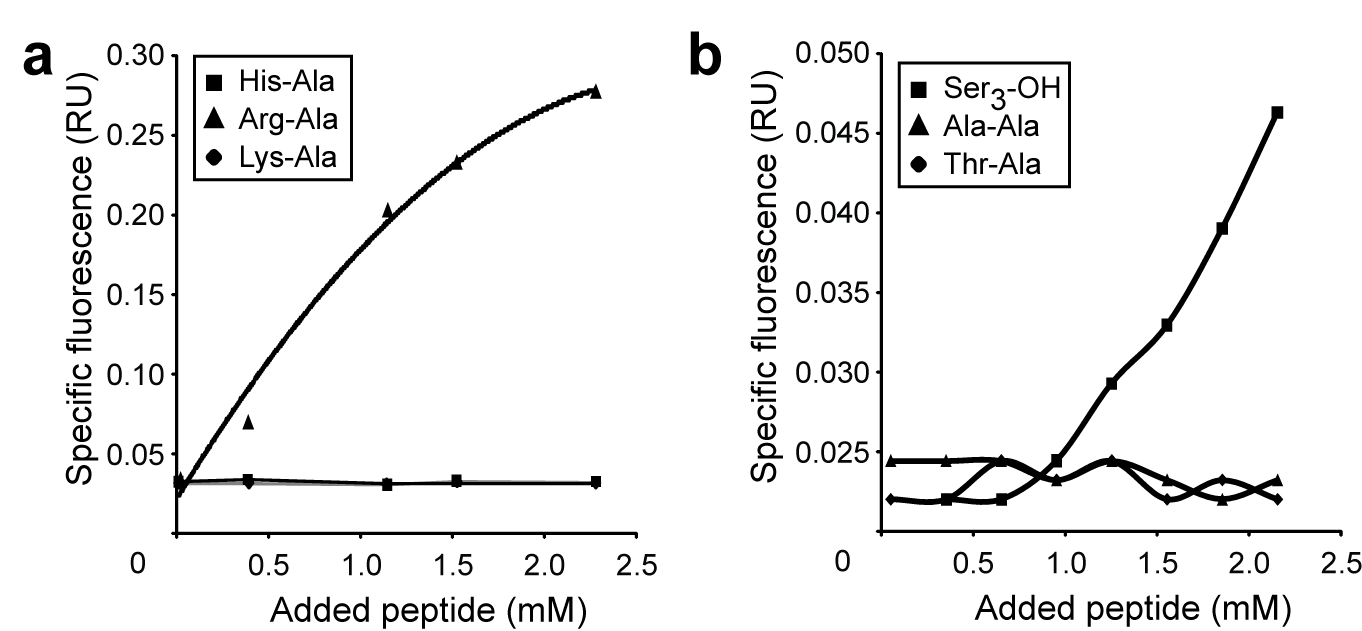
**

**Figure S3.** Peptide-dose response with *E. coli* pSenArg and *C. glutamicum* pSenSer. (**a**) *E. coli* pSenArg was cultivated in minimal medium and the specific peptide added at the given concentration. Addition of Arg-Ala results in fluorescent cultures, but this is not the case with His-Ala or Lys-Ala. (**b**) *C. glutamicum* pSenSer was cultivated in minimal medium and peptides added as shown. Ser-Ser-Ser results in fluorescent cultures, but this is not the case with Ala-Ala or Thr-Ala. In all cases Ala-Ala was included to give a total peptide concentration of 3 mM.


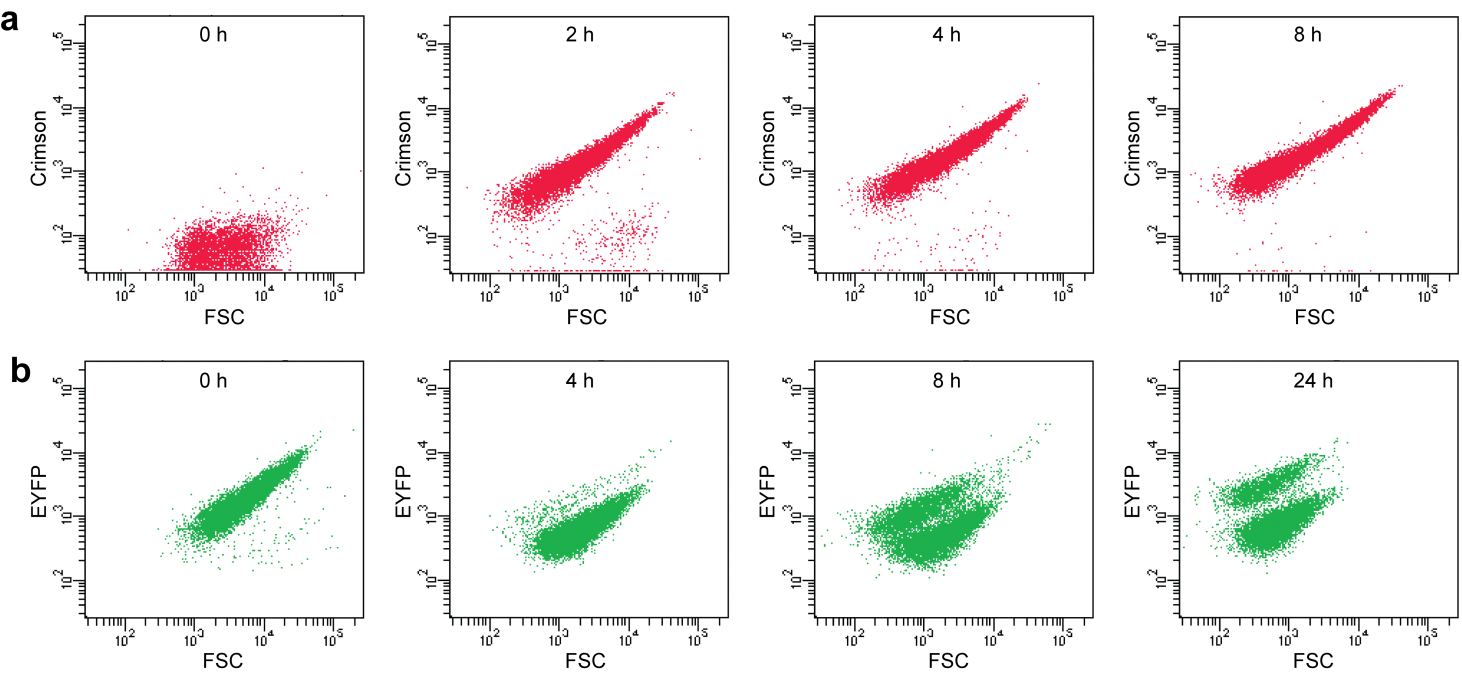


**Figure S4.** Development of Crimson and EYFP signals in mixtures of equal numbers of ATCC13032 and DM1728. To simulate the transfer of the mutant glycerol stock into minimal medium, the two cell types grown on BHI were mixed, glycerol was added and cells frozen. (**a**) Cells from the stock were diluted in minimal medium plus 0.1 mM IPTG. At the beginning of cultivation and 2, 4, and 8 hrs later, cells were assayed by flow cytometry for development of the Crimson signal. After 2 hrs, the majority of cells expressed Crimson, indicating active protein synthesis and thus living cells. (**b**) Cells were diluted as above and assayed at the beginning of culture and 4, 8, and 24 hrs later by flow cytometry for the EYFP signal. All cells derived from the complex medium exhibited high levels of fluorescence. After 4 hrs the majority showed reduced fluorescence, and after 8 hrs the signals specific for the two populations are apparent.

***
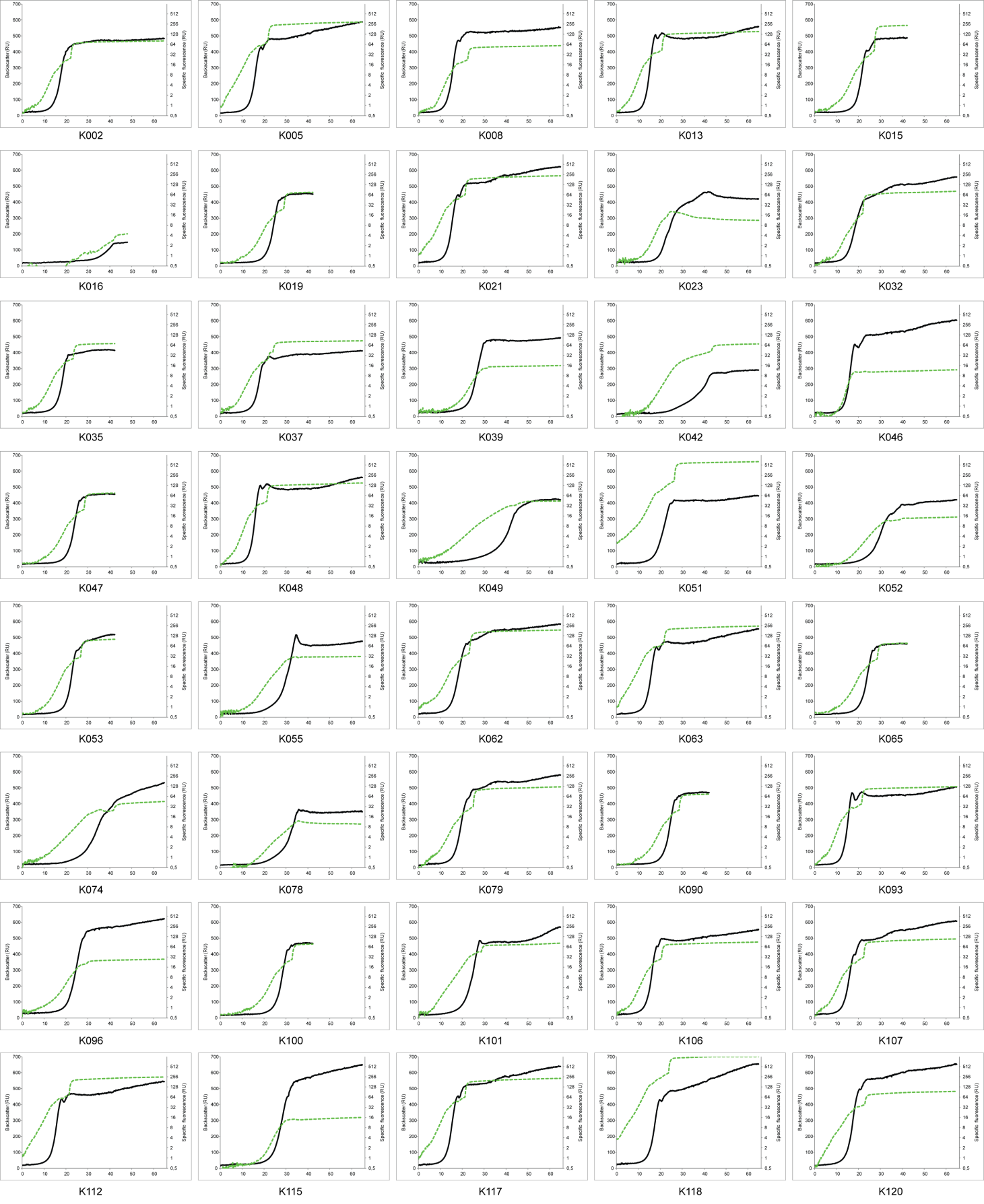
*** **Figure S5.** Growth curves and fluorescence for 40 mutant cultures. The BioLektor cultivation system was used (see Methods section) to follow the growth of 40 mutants in 0.75 ml cultures as measured by the backward scatter (black curve) and fluorescence at λ_ex_ 485 nm and λ_em_ 520 nm (green curve). Numbering of the different mutants is as given in Figure 4 of the main text.

**
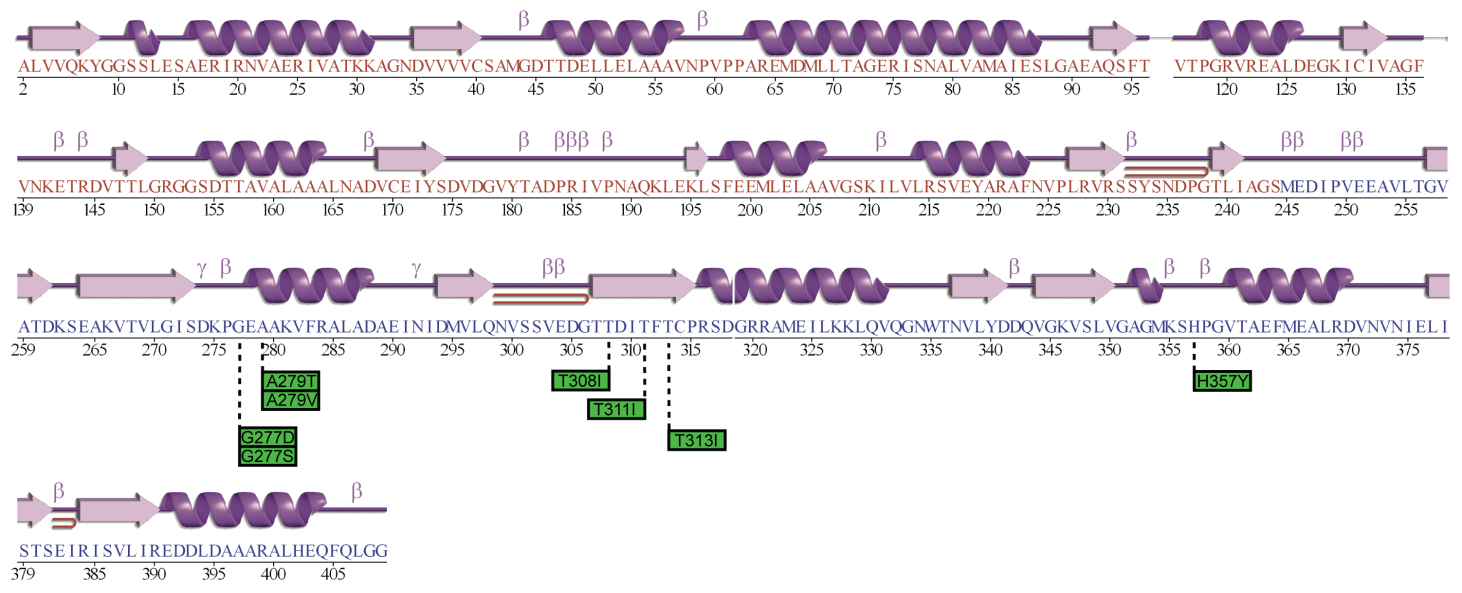
**

**Figure S6.** Structural presentation of LysC with the localization of mutations identified.

**REFERENCES**

1. Blombach B, Hans S, Bathe B, Eikmanns BJ: **Acetohydroxyacid synthase, a novel target for improvement of L-lysine production by Corynebacterium glutamicum.** *Appl Environ Microbiol* 2009, **75:**419-427.

2. Stolz M, Peters-Wendisch P, Etterich H, Gerharz T, Faurie R, Sahm H, Fersterra H, Eggeling L: **Reduced folate supply as a key to enhanced L-serine production by Corynebacterium glutamicum.** *Appl Environ Microbiol* 2007, **73:**750-755.

3. Grant SG, Jessee J, Bloom FR, Hanahan D: **Differential plasmid rescue from transgenic mouse DNAs into Escherichia coli methylation-restriction mutants.** *Proc Natl Acad Sci U S A* 1990, **87:**4645-4649.

4. Schäfer A, Tauch A, Jäger W, Kalinowski J, Thierbach G, Pühler A: **Small mobilizable multi-purpose cloning vectors derived from the Escherichia coli plasmids pK18 and pK19: selection of defined deletions in the chromosome of Corynebacterium glutamicum.** *Gene* 1994, **145:**69-73.

5. Ogawa N, McFall SM, Klem TJ, Miyashita K, Chakrabarty AM: **Transcriptional activation of the chlorocatechol degradative genes of Ralstonia eutropha NH9.** *J Bacteriol* 1999, **181:**6697-6705.

6. van der Ploeg JR, Iwanicka-Nowicka R, Kertesz MA, Leisinger T, Hryniewicz MM: **Involvement of CysB and Cbl regulatory proteins in expression of the tauABCD operon and other sulfate starvation-inducible genes in Escherichia coli.** *J Bacteriol* 1997, **179:**7671-7678.
